# Comparison of De-Torque and Failure Load Evaluation of Selective-Laser-Sintered CoCr, CAD-CAM ZrO, and Machined Implant Abutment/Restoration

**DOI:** 10.3390/bioengineering11050448

**Published:** 2024-04-30

**Authors:** Fahim Vohra, Rawan Alsaif, Rawaiz Khan, Ishfaq A. Bukhari

**Affiliations:** 1 Department of Prosthetic Dental Sciences, College of Dentistry, King Saud University, Riyadh 12485-6541, Saudi Arabia; alsaifrawanpros@gmail.com; 2 Engineer Abdullah Bugshan Research Chair for Dental and Oral Rehabilitation, College of Dentistry, King Saud University, Riyadh 12485-6541, Saudi Arabia; krawaiz@ksu.edu.sa; 3 Department of Pharmacology, College of Medicine, King Saud University, Riyadh 12485-6541, Saudi Arabia; bukhari1173@gmail.com

**Keywords:** computer-aided design, dental implants, lasers, printing, three-dimensional, zirconium

## Abstract

Aim: This study aimed to compare the torque loss, fracture load, compressive strength, and failure types of selective-laser-sintered cobalt chromium (SLM-Co-Cr), computer-aided design and computer-aided manufacturing zirconium oxide (CAD-CAM-ZrO), and machined titanium (Ti) implant abutments. Methods: Thirty endosseous dental implants were vertically embedded with machined Ti (control group), CAD-CAM-ZrO, and SLM-Co-Cr abutments. Abutment fabrication involved CAD-CAM milling and SLM technology. The de-torque assessment included preload reverse torque values (RTVs), cyclic loading, and post-RTVs using a customized protocol. Fracture load assessment employed ISO-14801 standards, and statistical analysis was conducted using ANOVA and Tukey Post hoc tests (*p* < 0.05). Results: In pre-load RTVs, SLM-Co-Cr showed the lowest mean torque loss (24.30 ± 2.13), followed by machined Ti (27.33 ± 2.74) and CAD-CAM-ZrO (22.07 ± 2.20). Post-load RTVs decreased for all groups. Fracture load and compressive strength were highest for SLM-Co-Cr, with significant differences among groups (*p* < 0.001). Fracture types included abutment failures in SLM-Co-Cr and machined Ti, while CAD-CAM-ZrO exhibited crown separation with deformation. Conclusion: SLM-Co-Cr-fabricated implant abutments exhibited superior stability and resistance to rotational forces, higher fracture loads, and greater compressive strength compared to CAD-CAM-ZrO and machined Ti.

## 1. Introduction

Selecting a suitable implant abutment is a pivotal aspect of achieving a successful outcome. Titanium (Ti) abutments have shown impressive survival rates [1], attributed to their outstanding biocompatibility and robust mechanical strength [2]. However, metallic abutments can sometimes lead to a grayish discoloration of the peri-implant mucosa, particularly in thin biotype soft tissues. Early esthetic designs using densely sintered alumina, though introduced, were linked to an increased risk of fracture [3,4]. Yttrium-stabilized zirconia with computer-aided design and computer-aided manufacturing (CAD-CAM) abutments demonstrated heightened mechanical strength compared to alumina and exhibited excellent biocompatibility similar to Ti [5]. However, it is essential to note that zirconia, being more brittle than Ti, has been associated with lower fracture resistance [6]. Systematic reviews comparing ceramic and metal abutments across both anterior and posterior areas supporting single-implant restorations and fixed partial dentures with external connections revealed no significant differences in technical complication rates or survival rates [1,7]. Despite these advancements, evidence suggests that even zirconia abutments may not fully integrate with natural teeth due to their bright white color, potentially causing blanching of the peri-implant mucosa [8].

There is a growing demand for high-strength substructures with a prevalence of long-spanning frameworks in prosthodontics for implant-supported prostheses [9]. The exploration of the mechanical behavior of diverse substructures is crucial for predicting the long-term success of dental prostheses [10]. Cobalt chromium (Co-Cr) serves as a prevalent base metal alloy employed in dental prostheses. Initially, Co-Cr alloys were predominantly utilized for the construction of metallic frameworks in removable prosthodontics. However, their application has now expanded to include fixed prostheses. The primary factor contributing to the growing use of Co-Cr is its cost-effectiveness [11]. Base metals have demonstrated their ability to fulfill the necessary physical properties for applications in high-functional-load scenarios, including long-span bridges and implant frameworks [12]. The conventional casting methods for Co-Cr frameworks tend to be challenging in terms of manufacturing. This challenge arises from their high melting temperatures, reduced ductility, and elevated hardness [13]. Additionally, the lost wax technique with casting may introduce unpredictability concerning the homogeneity of the resulting structure, thereby incorporating defects [14].

Alternates to casting fabrication include CAD-CAM manufacturing, which enhances control over both micro- and macrostructures in Co-Cr frameworks and ensures precise dimensions and management of materials during production [15]. Subtractive CAD-CAM techniques, such as milling, involve the reduction of a prefabricated Co-Cr block to the desired shape. However, milling techniques are associated with a notable amount of waste [16], and the high hardness of Co-Cr leads to significant wear on milling components when cutting a fully sintered Co-Cr block [17]. A relatively new fabrication method in the CAD-CAM category is three-dimensional printing (3DP), also known as additive manufacturing technology. It entails utilizing a low-energy layering process and circumvents the physical challenges associated with the hard machining of alloys. It reduces component stress but also significantly minimizes material waste [16,17]. Unlike milling and casting techniques, which are constrained by the size of the block or investment chamber, additive manufacturing can be employed for creating single-piece maxillofacial models or metallic prostheses [18]. For Co-Cr alloy laser sintering (LS), a bed of Co-Cr powder is sintered layer by layer to form a larger structure. Although both 3D laser sintering and soft milling (SM) techniques provide alternative low-stress manufacturing for Co-Cr prosthetic frameworks, there is a scarcity of published studies reporting on the physical properties of LS and SM manufacturing methods using standardized methodologies [12].

The success of implant-supported prostheses depends on the implant-abutment junction stability, which is directly related to the amount of preload generated during insertion and maintained over time. The limitations of abutment fabrication techniques can lead to inferior torque maintenance in comparison with the preformed machined abutments. In a previous study, it was suggested that the original abutments showed less torque loss than the three copy abutments [19]. This result may be due to the limitations of the manufacturing tolerance and accuracy of milling machines. Limited evidence is available on the loss of torque and strength of laser-sintered CoCr abutments compared to conventional machined or CAD-CAM implant abutments in the present literature.

Therefore, the present study aimed to compare the torque loss, fracture load, compressive strength, and failure types of SLM-Co-Cr, CAD-CAM zirconium oxide (CAD-CAM-ZrO), and machined Ti implant abutments. The null hypothesis posited that there is no difference in the mechanical properties between specimens produced using either manufacturing method.

## 2. Materials and Methods

### 2.1. Sample Size Calculation

To determine the appropriate sample size, a power analysis was conducted using data from a similar investigation [20]. Utilizing parameters of 80% power, a 95% confidence interval (α = 0.05), and an effect size of 0.60, it was advised that a minimum sample size of 8 specimens be considered. Considering potential sample failures, a decision was made to include 10 samples per group.

### 2.2. Specimen Fabrication and Research Groups

Thirty endosseous dental implants (Ø 4.0 mm × 10 mm, Dentium Co., Seoul, Korea) (4.0 mm × 10 mm) were vertically embedded in polymethyl methacrylate resin (Major Ortho™, Torino, Italy) using a Ney surveyor (Dentsply-Sirona Inc., York, PA, USA), with two implant threads exposed. Ten machined Ti alloy abutments (Dentsply-Sirona Inc., York, PA, USA), (4.5 mm × 10 mm; soft tissue) were used as controls (machined Ti). Additionally, ten ZrO abutment crowns were produced through CAD-CAM milling (Y-TZP-CAD-CAM), while ten Co-Cr alloy abutment crowns were manufactured using SLM technology (SLM-Co-Cr).

The ZrO abutment crowns were crafted using the ZirconZahn system (An der Ahr, Gais, Italy). A scan-marker for the Dentium System (An der Ahr, Gais, Italy) with a 4.8 mm diameter was scanned using the Zirkonzahn optical scanner (An der Ahr, Gais, Italy) (S600 ARTI). The abutment platform was selected from the Zirkonzahn implant digital library for Dentium superline abutment, and the coronal part of the crown was designed with standard mandibular second premolar dimensions from the library. The virtual implant abutment crown model in standard tessellation language (STL) file format was then saved. Subsequently, ten SLM-Co-Cr screw-retained crowns were produced using the designed STL file.

**Figure 1:**
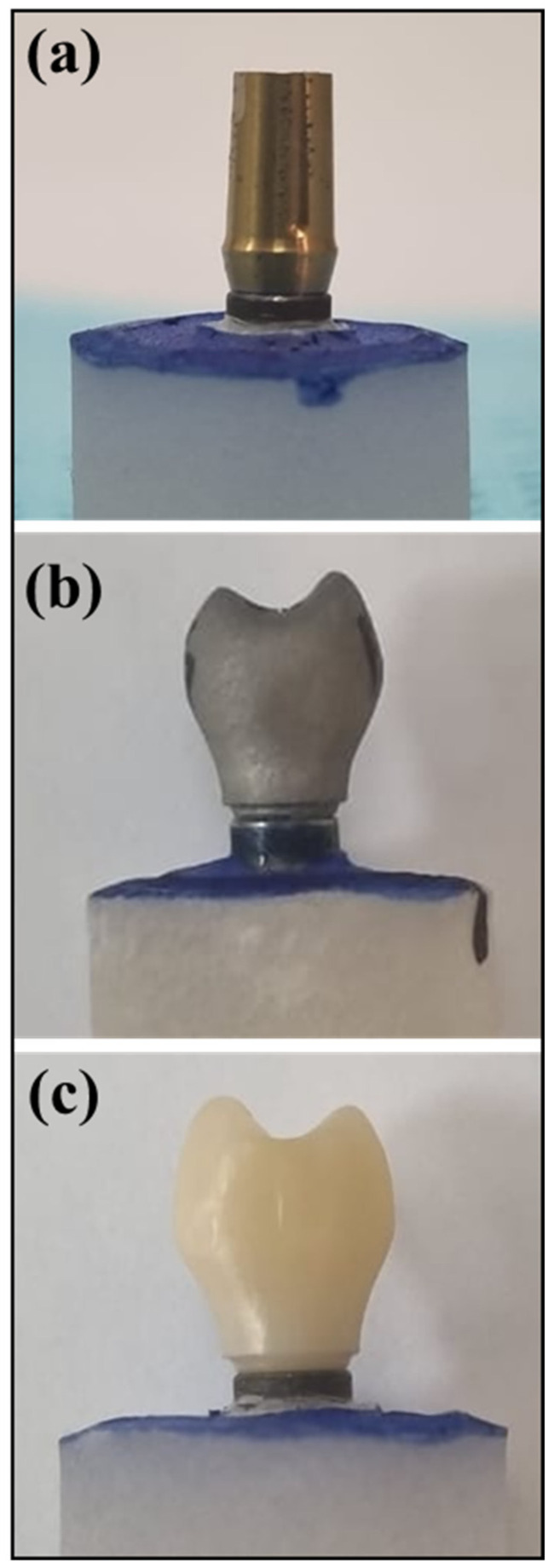
Implant abutment samples. (**a**) Machined titanium preformed abutment. (**b**) SLM-CoCr abutment fabricated using the selective laser melting technique (3D printing). (**c**) CAD-CAM-ZrO abutment fabricated using computer-aided design and computer-aided manufacturing method with zirconium oxide material.

The abutment model was transferred to the concept laser machine (Mlab cusing metal laser melting system; GE Additive company, Boston, MA, USA) using Co-Cr alloys (Starbond Easy Powder; Scheftner GmbH, Mainz, Germany). Additionally, Sisma (3D laser metal fusion technology; Vicenza, Italy) was employed using Ti 6Al–4V powder grade 23 (TI64GD 23, LPW Technology, Lomdon, UK) following specific recommended parameters. The resulting implant abutment/crown, including the fabricated CAD-CAM-ZrO and SLM-Co-Cr as well as the control (machined Ti), is visually presented in Figure 1a–c.

### 2.3. Abutment De-Torque Assessment

The specimens were randomly distributed and affixed using a customized holder before initiating the torquing procedures. Each set of 10 samples underwent individual steps of preload reverse torque values (RTVs), cyclic loading, and post-RTVs, with the entire sequence completed before moving on to the next group. The implant restorations were initially secured to the implants at 30 Ncm and retorqued after 15 min, as per the manufacturer’s recommendation, utilizing a Tohnichi BTGE digital torque gauge (Tohnichi Mfg, Tokyo, Japan). Preload-RTV measurements were conducted after 2 min, and all measurements were executed by a single trained operator. Before cyclic loading, the implant restorations were again retorqued to 30 Ncm. In the case of the machined Ti group abutments, crowns made of Co-Cr using selective laser sintering with standard mandibular second premolar dimensions were prepared. A layer of Teflon was applied, and the crowns were cemented, with the access blocked using Teflon material. Subsequently, the crowns were cemented with temporary zinc oxide eugenol cement (Temp-Bond; Kerr Corporation, Orange, CA, USA). The resin-mounted implant block was then positioned in a customized mold consistent with the mold employed in the chewing simulator machine.

Following a 24 h cementation period, cyclic loading was conducted using a CS4 chewing simulator machine (SD Mechatronic, Feldkirchen, Westerham, Germany). All assemblies were subjected to a consistent load of 5 kg, undergoing 300,000 cycles at a frequency of 1 Hz. The antagonists, crafted from composite material (f Z350 XT, 3M ESPE, St. Paul, MI, USA), were immersed in distilled water filled up to 2 mm above the platform. Each group underwent a week-long exposure to cyclic loading. During cyclic loading, the samples exposed to chewing simulation were immersed in a normal saline solution. Postload-RTV assessments were conducted immediately after removing the samples from the chewing simulator. The differences between pre-RTVs and post-RTVs were calculated and denoted as RTV difference (RTD).

### 2.4. Fracture Load Assessment

**Figure 2:**
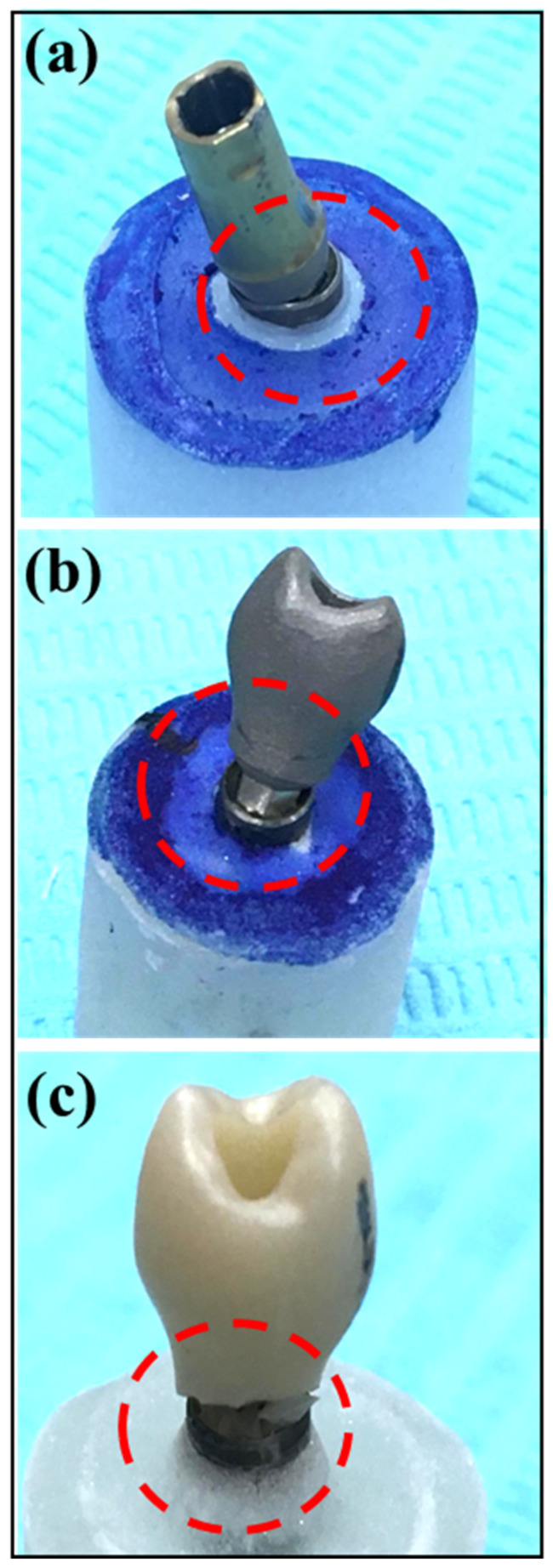
Implant abutment samples after fracture. The red circle represents the fracture/failure site (implant–abutment connection) among the different abutment types: (**a**) SLM-CoCr; (**b**) machined Ti; and (**c**) CAD-CAM-ZrO.

The implant abutment complex (IAC) of samples in all study groups underwent exposure to static controlled loads at a 30° angle to the long axis, following ISO-14801 standards. The loads were applied using a universal testing machine (INSTRON 5965 Material Testing System, Norwood, MA, USA) at a rate of 1.0 mm/min until failure. To ensure even load distribution over the restoration, a 0.5 mm thick tin foil (Dentaurum; GmbH & Co. KG, Ispringen, Germany) was interposed between the blunt end indenter metal probe and the restoration. A failure of the restoration was defined as either a noticeable fracture of the implant crown, abutment, or restoration or a reduction in maximum load by 20% during the load test, even in the absence of detected fractures (Figure 2). The maximum load at failure and the compressive force were recorded for each specimen, and means with standard deviations within groups were determined. Fracture patterns were also categorized as follows: Type-I: crown failure; Type-II: screw or abutment failure; Type-III: crown separation from substructure with deformation; and Type-IV: crown separation from substructure with no deformation.

### 2.5. Statistical Analysis

The data were subjected to analysis using the Statistical Package for the Social Sciences (SPSS Version 20, IBM Microsoft, New York, NY, USA). The normality of the data was evaluated through the Kolmogorov–Smirnov test. Means and standard deviations of variables were compared utilizing analysis of variance (ANOVA), and Tukey post hoc comparison tests were applied, with the significance level set at *p* < 0.05.

## 3. Results

### 3.1. Torque Loss Outcomes

**Figure 3:**
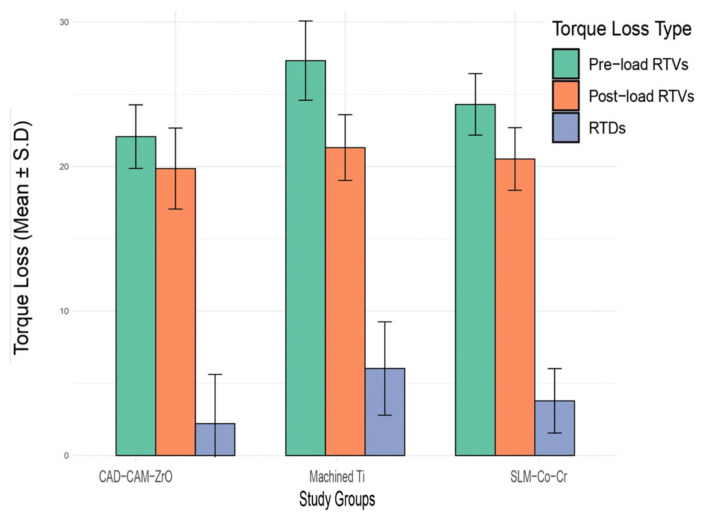
Comparison of mean torque loss among study groups. RTV, reverse torque value; RTD, reverse torque difference.

Table 1 shows the mean torque loss among three study groups (Figure 3). In the pre-load RTVs, the SLM-Co-Cr group exhibited a mean torque loss of 24.30 ± 2.13, the machined Ti group showed 27.33 ± 2.74, and the CAD-CAM-ZrO group showed 22.07 ± 2.20. Post-load RTVs revealed a decrease in torque loss for all groups, with values of 20.52 ± 2.23, 21.31 ± 3.23, and 19.86 ± 3.40, respectively. The RTD demonstrated similar trends, with SLM-Co-Cr showing 3.78 ± 2.17, machined Ti exhibiting 6.02 ± 2.27, and CAD-CAM-ZrO showing 2.21 ± 2.80. Statistical analysis indicated significant differences among the study groups for pre-load RTVs (*p* < 0.001) and post-load RTVs (*p* = 0.019), while post-load RTDs showed no significant differences (*p* = 0.086).

### 3.2. Fracture Load and Compressive Strength Outcomes

Table 2 shows the fracture loads and compressive stress values for the study groups. In terms of fracture load, SLM-Co-Cr exhibited the highest mean value at 700.063 ± 110.10 N, followed by machined Ti with 533.559 ± 69.37 N and CAD-CAM-ZrO with 419.097 ± 44.80 N. Correspondingly, compressive strength values followed a similar trend, with SLM-Co-Cr having the highest mean compressive strength at 55.709 ± 7.56 MPa, machined Ti at 42.459 ± 10.60 MPa, and CAD-CAM-ZrO at 30.716 ± 9.04 MPa. Statistical analysis indicated significant differences among the study groups for both fracture loads (*p* < 0.001) and compressive stress (*p* = 0.018).

### 3.3. Fracture Type Outcomes

Most fractures among SLM-CoCr (100%) and machined Ti (100%) samples were abutment failures (Type-II). However, all failures among CAD-CAM-ZrO samples were crown separation with deformation (Type-III) (100%). Most failures (machined Ti and SLM-CoCr) resulted in bending rather than breaking/fracture of the abutment screws. The CAD-CAM-ZrO samples showed complete fracture of the ceramic crown detachment from the abutment. This indicated the catastrophic clinical failure of machined Ti and SLM-CoCr as screw bending would result in non-retrieval of the abutment and failure of unscrewing of the abutment screw. However, the ZrO abutment fracture would allow for comparatively simple retrieval of the abutment and replacement with a new abutment/crown restoration.

## 4. Discussion

This study aimed to compare the torque loss, fracture load, compressive strength, and failure types of SLM-CoCr, CAD-CAM-ZrO, and machined Ti implant abutments. In pre-load RTVs, the SLM-Co-Cr group exhibited the lowest mean torque loss, while the post-load RTVs showed a decrease in torque loss for all groups. Fracture load and compressive strength values were highest for SLM-Co-Cr. Statistical analysis revealed significant differences among the groups for pre-load and post-load RTVs, fracture loads, and compressive stress. Furthermore, the fracture type outcomes indicated distinct failure patterns, with abutment failures in SLM-Co-Cr and machined Ti samples, while CAD-CAM-ZrO samples exhibited crown separation with deformation.

One noteworthy finding of this study was observed in the torque loss measurements during pre-load-RTA. The SLM-Co-Cr group demonstrated the lowest mean torque loss, suggesting superior stability and resistance to rotational forces compared to the CAD-CAM-ZrO and machined Ti groups. Interestingly, post-load RTA revealed a general decrease in torque loss across all groups. It can be inferred that the observed decrease in torque loss across all groups in the post-load RTA may be attributed to potential adaptive responses or settling effects after the application of load. This phenomenon has been documented in the context of screw stability and torque loss in dental implant systems [21].

The assessment of fracture load and compressive strength yielded compelling results, with the SLM-Co-Cr group exhibiting the highest values for both parameters. This outcome underscores the robust mechanical properties of SLM-Co-Cr implant abutments, suggesting their potential suitability for applications requiring high load-bearing capacity and resistance to fractures. This study’s findings align with those of existing research on the mechanical properties of different implant abutment materials. For instance, a study on the fracture resistance and wear induced by single-unit screw-retained CAD components fabricated by various CAM methods reported that the SLM material showed increased wear but presented higher mechanical fracture load measurements compared to other materials [22]. Additionally, a study on the fracture resistance of different types of CAD-CAM-ZrO abutments under static loading also provides insights into the mechanical properties of these materials, which are relevant to the observed fracture load and compressive strength values in the present study [23]. It is noteworthy that although the coronal geometry of the machined Ti abutment type was different from the SLM-CoCr CAD-CAM-ZrO abutment, the abutment-to-implant connection geometry for all three abutments was similar in dimension. In addition, the fracture load outcomes showing higher load outcomes for machined Ti compared to CAD-CAM-ZrO suggest that the coronal geometry of the machined Ti abutment did not influence the fracture load. This also highlights the fact that fractures for implant-supported restorations within abutments take place at the implant–abutment connection at the hexagon. The three abutment types that were compared in the present study were chosen due to them being a treatment option for the same implants in different clinical situations based on the location of the implant, patient desire for esthetics, implant angulation, soft tissue height around the implant, and restorative space. In patients with anterior implants, either a CAD-CAM-ZrO or a machined Ti abutment with a ceramic crown would be the choice due to high esthetic demand based on the implant angulation. In case of adequate implant angulation, a CAD-CAM-ZrO would be indicated; however, if the screw access channel exits at the buccal aspect of the crown, then a machined Ti and a cement-retained crown would be the esthetic choice. In addition, in a posterior tooth case of limited soft tissue height and or limited restorative space, a screw-retained CAD-CAM-ZrO or SLM-CoCr abutment/restoration would be the option of choice based on the patient’s esthetic demands.

The statistical analysis of the data revealed significant differences among the groups for pre-load and post-load-RTAs, fracture loads, and compressive stress. This study’s findings are in line with the growing body of research focused on the mechanical properties of implant abutments. For example, a study on the mechanical properties and marginal fit of prefabricated versus customized implant abutments provides relevant insights for the clinical selection of implant abutments, which is in line with the practical implications of the present study’s findings [24]. Additionally, research on the effect of platform-switching implants and different abutment materials on the stress distribution of implant-supported restorations contributes to the understanding of the mechanical behavior of implant components, further supporting the significance of the findings for clinical applications [25].

This study delved into the types of failures exhibited by the abutments, revealing distinctive patterns. Abutment failures were prominent in the SLM-Co-Cr and machined Ti groups, while CAD-CAM-ZrO samples demonstrated crown separation with deformation. This study’s findings are in line with the existing literature on the failure modes of implant abutments. For instance, a study on the failure modes and survival of anterior crowns supported by narrow implant systems reported that the failure mode predominantly involved abutment and/or abutment screw fracture, highlighting the relevance of understanding specific failure patterns for different implant components [26]. Additionally, research on the effect of fatigue loading and the failure mode of different ceramic implant abutments provides insights into the failure modes of specific abutment materials, which is relevant to the observed distinctive failure patterns in the present study [27]. In the present study, most failures (machined Ti and SLM-CoCr) resulted in bending rather than fracture of the abutment screws. ZrO samples showed complete fracture of the ceramic crown detachment from the abutment, highlighting the potential for catastrophic clinical failure of machined Ti and SLM-CoCr as screw bending would result in non-retrieval of the abutment/screw. Comparatively, a ZrO abutment fracture would allow for comparatively simple retrieval of the abutment and replacement with a new abutment/crown restoration.

This study’s findings demonstrate the advantages of increased strength and failure resistance in the clinical application of SLM-CoCr and machined Ti abutments in comparison with ZrO abutments/restorations. In addition, the fracture loads observed for ZrO showed high values and potentially allow for good clinical fracture resistance. In addition, the potential for screw loosening due to clinical function among the three different implant abutment restorations is comparable based on in vitro observations in the present study. While the present in vitro study discussed valuable insights into the mechanical properties of various implant abutment materials, it is crucial to acknowledge several limitations to interpret the findings cautiously and guide future research. This study’s applicability to clinical scenarios is restricted due to its in vitro nature as it did not replicate the biological responses and patient anatomy variations encountered in vivo [27]. Thus, caution is warranted in translating these findings to clinical practice, necessitating further investigations through clinical trials. This study’s sample size (10 specimens per group) was determined through a power analysis, suitable for the statistical analyses performed. However, this size may not fully capture the inherent variability in biological and mechanical responses. Larger sample sizes would enhance the robustness of the study and provide a more comprehensive understanding of material behaviors [24]. Simplified loading conditions in this study may not have fully replicated the intricate forces experienced by dental implants in real-world situations. The chewing simulator’s controlled cyclic loading, while useful, might not entirely mimic the complex forces during activities including mastication and speech [24]. This study’s focus on specific implant abutment materials and designs overlooked potential variations within each material category, such as fabrication techniques and surface treatments [22]. A more detailed exploration of these factors would contribute to a nuanced understanding of material performance. Extending this study to include fatigue testing under dynamic conditions could offer a more comprehensive assessment of material durability. Lastly, this study did not consider the potential influence of long-term exposure to oral conditions, such as corrosion, wear, or biofilm formation. Future research should include long-term studies to assess the stability and reliability of these materials in realistic clinical contexts. In addition, assessing the impact of lateral loads and analyzing biofilm formation on different surfaces (ZrO, SLM CoCr, and Ti alloys) and the corrosion characteristics of the differently fabricated abutments and materials would provide insights into the clinical performance and behavior of the printed, milled, and machined abutments. Addressing these limitations will contribute to a more comprehensive understanding of the performance and applicability of implant abutment materials in clinical settings.

## 5. Conclusions

SLM-Co-Cr fabricated implant abutments exhibited superior stability and resistance to rotational forces, higher fracture loads, and greater compressive strength compared to CAD-CAM-ZrO and machined Ti.

## Figures and Tables

**Table 1:** Comparison of mean torque loss among study groups.

Preload RTVs Postload RTVs RTDs				
Groups	N	Mean + SD	Mean + SD	Mean + SD
SLM-CoCr	8	24.30 + 2.13 ^a^	20.52 + 2.23 ^a^	3.78 + 2.17 ^a^
Machined Ti	8	27.33 + 2.74 ^b^	21.31+ 3.23 ^a^	6.02 + 2.27 ^b^
CAD-CAM-ZrO	9	22.07 + 2.20 ^a^	19.86 + 3.40 ^a^	2.21 + 2.80 ^a^
*p*-value		<0.001	0.019	0.086

Different superscript small alphabets in same column denote significant difference (*p* < 0.05). RTV, reverse torque value; RTD, reverse torque difference.

**Table 2:** Means and standard deviations of fracture loads (N) and compressive stress (MPa) among study groups.

Groups	N	Fracture Load (N) Mean + SD	Compressive Strength (MPa) Mean + SD
SLM-CoCr	8	700.063 + 110.10 ^a^	55.709 + 7.56 ^a^
Machined Ti	8	533.559 + 69.37 ^b^	42.459 + 10.60 ^b^
CAD-CAM-ZrO	9	419.097 + 44.80 ^c^	30.716 + 9.04 ^c^
*p*-value		<0.001	0.018

Different superscript small alphabets in same column denote significant difference (*p* < 0.05).

## Data Availability

All the data are included in the manuscript.
